# Cyclodextrins as Chiral Selectors in Capillary Electrophoresis—Recent Developments in Understanding Analyte Complexation and Applications in Pharmaceutical Analysis

**DOI:** 10.1002/elps.70116

**Published:** 2026-06-01

**Authors:** Gerhard K. E. Scriba

**Affiliations:** ^1^ Department of Pharmaceutical/Medicinal Chemistry Friedrich Schiller University Jena Jena Germany

**Keywords:** chiral recognition mechanism, cyclodextrin, enantiomeric purity, enantioseparation, pharmaceutical analysis

## Abstract

CDs, cyclic oligosaccharides consisting of α‐1,4 linked d‐glucopyranose units, have been applied in many areas, such as food, cosmetics, environmental, agriculture, textile, pharmaceutical, and chemical industries, due to their ability to form guest–host complexes. Because CDs are chiral compounds, they have also been successfully used as chiral selectors in analytical enantioseparation techniques, especially CE. In the last decades, multidisciplinary approaches have been performed, combining CE with NMR spectroscopy, molecular modeling, and other techniques in order to understand aspects of chiral recognition underlying enantioseparations. The present review focuses on such mechanistic studies published between January 2023 and March 2026 in addition to the application of CD‐mediated CE enantioseparations to the determination of the enantiomeric purity of drug substances as well as the analysis of pharmaceutical formulations.

## Graphical abstract

Cyclodextrins are the most often applied chiral selectors in CE enantioseparations. In combination with NMR spectroscopy and molecular modeling, studies for an understanding of chiral recognition underlying enantioseparations have been performed. The review summarizes recent advances in this field as well as the application of CD‐mediated enantioseparations in pharmaceutical analysis.

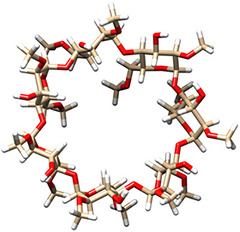

**Abbreviations**
2,3‐DM‐β‐CD: *heptakis*(2,3‐di‐*O*‐methyl)‐β‐CD
2,6‐DM‐β‐CD: *heptakis*(2,6‐di‐*O*‐methyl)‐β‐CD
2‐M‐β‐CD: *heptakis*(2‐*O*‐methyl)‐β‐CD
3,6‐DM‐β‐CD: *heptakis*(3,6‐di‐*O*‐methyl)‐β‐CD
3‐M‐β‐CD: *heptakis*(3‐*O*‐methyl)‐β‐CD
6‐M‐β‐CD: *heptakis*(6‐*O*‐methyl)‐β‐CD
ATP: analytical target profile
CM‐β‐CD: carboxymethyl‐β‐CD
COSY: correlated spectroscopy
CPPs: critical process parameters
CQA: critical quality attributes
DES: deep eutectic solvent
DFT: density functional theory
Dns: dansyl
DoE: design of experiments
DS: degree of substitution
EMO: enantiomer migration order
HDA‐β‐CD: *heptakis*(2,3‐di‐*O*‐acetyl)‐β‐CD
HMA‐β‐CD: *heptakis*(2‐*O*‐methyl‐3‐*O*‐acetyl)‐β‐CD
HMBC: heteronuclear multiple bond correlation
HP‐β‐CD: 2‐hydroxypropyl‐β‐CD
HSQC: heteronuclear single quantum coherence
HS‐β‐CD: *heptakis*(6‐*O*‐sulfo)‐β‐CD
ICH: International Council for Harmonisation of Technical Requirements for Pharmaceuticals for Human Use
IL: ionic liquid
MODR: method operable design region
M‐β‐CD: methylated β‐CD
NOE: nuclear Overhauser effect
NOESY: nuclear Overhauser enhancement spectroscopy
OFAT: one factor at a time
PDES: polymeric deep eutectic solvent
PS: pattern of substitution
QbD: quality by design
ROESY: rotating frame Overhauser enhancement spectroscopy
SBE‐β‐CD: sulfobutylether‐β‐CD
SUPRADES: supramolecular deep eutectic solvent
S‐β‐CD: sulfated β‐CD
S‐γ‐CD: sulfated γ‐CD
TM‐β‐CD: *heptakis*(2,3,6‐tri‐*O*‐methyl)‐β‐CD
TOCSY: total correlation spectroscopy

## 1 Introduction

CDs are cyclic oligosaccharides with a lipophilic cavity and a hydrophilic exterior. The compounds were discovered by the French chemist Antoine Villers in 1891 as crystalline byproducts in incubations of potato starch in the presence of a *Bacillus* species [1]. CDs are nowadays produced by digestion of starch with cyclodextrin glycosyl transferase (EC 2.4.1.19) obtained from various *Bacillus* strains [2]. The term “cyclodextrin” was first proposed by the German chemist Friedrich Cramer in 1949 [1] and later adopted by IUPAC [3].

The ability of CDs to form (primarily) inclusion complexes with organic and inorganic molecules, which affects many properties of the complexed compounds, including solubility, stability, and taste or odor, has led to widespread technological applications in pharmaceutical, food, cosmetic, chemical, or textile industries [4, 5, 6, 7, 8, 9, 10]. Because the complex formation occurs in a stereoselective manner, CDs have been applied as chiral selectors to the analysis of stereoisomers in separation techniques, including GC [11, 12, 13], HPLC [13, 14, 15, 16], super/subcritical fluid chromatography [17, 18], and especially CE [19, 20, 21, 22, 23, 24, 25, 26, 27, 28, 29]. Most recently, CDs were also used as chiral auxiliaries for the separation of stereoisomers in ion mobility mass spectrometry [30, 31, 32].

The first use of CDs as chiral selectors in CE has been reported in the early 1990s by the group of Shigeru Terabe on the enantioseparation of dansyl (Dns) dl‐amino acids by CD‐mediated MEKC [33] as well as by Salvatore Fanali, who separated the enantiomers of terbutaline and propranolol in the presence of *heptakis*(2,6‐di‐*O*‐methyl)‐β‐CD (2,6‐DM‐β‐CD) or β‐CD [34]. Significant contributions in this field were provided by Bezhan Chankvetadze, who applied sulfobutylether β‐cyclodextrin (SBE‐β‐CD) to the separation of the enantiomers of basic drugs as well as the neutral analyte thalidomide in 1994 [35] and published numerous papers on the CD‐mediated CE enantioseparations, including applications, fundamental, and mechanistic studies, just to name a few (see, e.g., Refs. [20, 36, 37, 38, 39, 40, 41]).

The present review summarizes recent developments in the understanding of complex formation between CDs and chiral analytes as basis for CE enantioseparations as well as the application of CDs as chiral selectors in CE for pharmaceutical analysis. Because the CDs act as a pseudo‐stationary phase, the technique is also referred to as EKC. The summary focuses on the literature published between 2023 and March 2026. For earlier reviews on the structure of analyte‐CD complexes see, e.g., Refs. [20, 22, 27, 41, 42, 43, 44, 45, 46], for recent reviews of CD‐mediated CE enantioseparations of pharmaceutical drugs see, e.g., Refs. [22, 23, 47, 48, 49, 50, 51, 52, 53, 54].

## 2 Cyclodextrin Structure and Solute Complexation

### 2.1 Structural Aspects

CDs are composed of d‐glucopyranose units linked via α‐1,4 glycosidic bonds. The most abundant native CDs obtained by cyclodextrin glycosyl transferase incubations differ in the number of d‐glucopyranose units and, consequently, cavity size. α‐CD is composed of six d‐glucopyranose units, β‐CD of seven, and γ‐CD of eight. CDs composed of 9 to up to 31 d‐glucopyranose units, so‐called large ring CDs, have been isolated from incubations [55, 56], but the CE enantioseparation ability of such CDs was only scarcely studied [57].

In the native CDs, the d‐glucopyranose moieties are in the ^4^C_1_ chair conformation, resulting in a relatively rigid almost circular structure with a hydrophilic outside and a hydrophobic cavity. The 2‐ and 3‐hydroxyl groups are oriented toward the secondary, wider rim of the CDs, whereas the 6‐OH groups form the narrower, primary rim. The interior of the cavity is lined by the axial C(3)‐H and C(5)‐H as well as the glycosidic oxygen atoms, rendering it lipophilic. On the basis of the radii formed by the C‐1, C‐2, and C‐6 carbon atoms, the structure is typically described in the literature as a truncated conical cylinder [58]. A recent modeling study on the hydration of the CD cavity suggested to consider the positions of the oxygen atoms, that is, the glycosidic oxygens as well as the oxygens of the primary and secondary hydroxyl groups to define the geometry of the CD cavity [58]. Because of the inward protrusion of the glycosidic oxygens into the cavity, the inner diameter formed by these oxygens is smaller than the outer primary and secondary rims so that the structure of a conical hourglass was suggested. For further details on structural and physicochemical properties of the native CDs, see, for example, [25, 59, 60].

**FIGURE 1:**
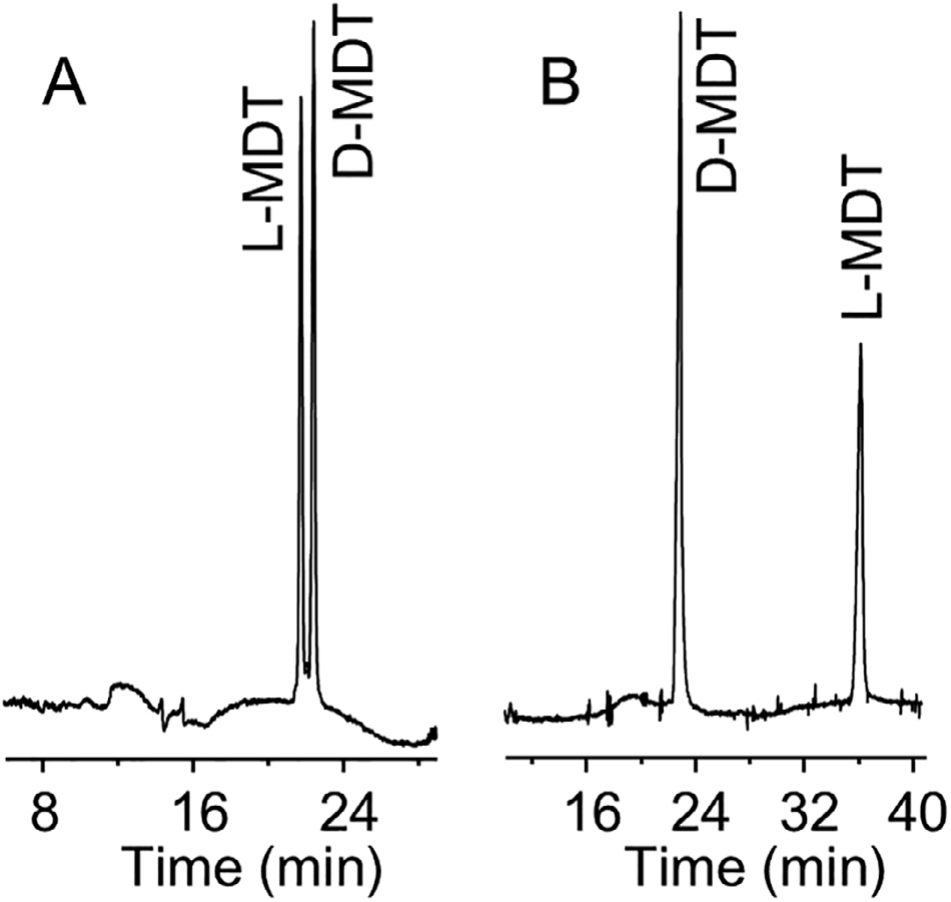
Comparison of CE enantioseparations of the enantiomers of medetomidine using (A) HS‐β‐CD and (B) S‐β‐CD as chiral selector. Experimental conditions: 40/50.2 cm, 50 µm i.d. fused silica capillary; 50 mM sodium phosphate buffer, pH 2.5 containing 20 mM of the CD; 20°C; detection at 200 nm; sample concentration 100 µg mL^−1^ dexmedetomidine (D‐DMT) and 50 µg mL^−1^ levomedetomidine (L‐MDT). Adapted from Ref. [69] with permission from Elsevier, 2018.

The hydroxyl groups of the d‐glucopyranose units can be derivatized, introducing various types of substituents. This results in a large number of derivatives [61, 62, 63, 64, 65], many of which are commercially available. The OH groups possess different reactivities. For example, the 6‐OH group is the least acidic one, and the 2‐OH group is the most acidic one. On the other hand, the 6‐OH group is more accessible, especially for spacious reagents. Substitution of the 3‐OH group is most difficult. Thus, various site‐ and regio‐specific reactions can be applied in order to obtain CDs with specific substituents in a defined position, derivatives with different substituents at certain positions, or derivatives with a substituent in a position of a single glucopyranose unit [19]. Native CDs and their derivatives can be grouped according to the “CD basis,” that is, α‐CDs, β‐CDs, and γ‐CDs, or according to the charge introduced by the substituents, for example, neutral (uncharged) and charged CDs. Another classification is based on the fact if substituents have been introduced only in a certain position of the molecule, so‐called single isomer CDs, or if derivatization of the OH groups occurred randomly, resulting in the so‐called randomly substituted CD derivatives. The latter are composed of a large number of different CDs, varying in the number of substituents (substitutional isomers characterized by the degree of substitution, DS) as well as their positions (positional isomers, characterized by the pattern of substation, PS). The exact composition of these CDs with regard to the numerous isomers present is not known. A theoretical study has concluded that for a CD with a single substituent type, considering the derivatives with single modifications at C‐2, C‐3, and C‐6, derivatives with two modifications (at Positions 2 and 3, 2 and 6, as well as 3 and 6), as well as derivatives with modifications at all three hydroxy groups and the native CD, will amount to 43 800 unique structures in the case of α‐CD, 299 600 structures in the case of β‐CD, and 2 097 684 structures in the case of γ‐CD [66]. Consequently, randomly substituted CDs cannot be considered a well‐defined molecule, and the outcome observed in an application, such as an enantioseparation, is the averaged effect over all substitutional and positional isomers present in the mixture as has been also theoretically derived in a migration model by Dubsky et al. [67, 68]. Nonetheless, effective enantioseparations often with enhanced selectivity compared to single isomer CDs can be achieved with randomly substituted CDs as illustrated in Figure 1 for the separation of the enantiomers of medetomidine in the presence of the single‐isomer CD *heptakis*(6‐*O*‐sulfo)‐β‐CD (HS‐β‐CD) and randomly substituted sulfated β‐CD (S‐β‐CD) [69]. In addition to different separation selectivities, opposite enantiomer migration order (EMO) was observed. A similar result was obtained for a series of oxazolidinone analogs when comparing S‐β‐CD and HS‐β‐CD as chiral selectors in CE [70]. These studies as well as others illustrate that the overall effect of the CD isomer mixture in a randomly substituted CD can differ from the chiral recognition of analytes by single isomer CDs.

In contrast to single‐isomer CDs, analytical characterization of randomly substituted CDs is complex [71]. Techniques, such as HPLC or CE, can separate substitution isomers according to their DS and in some cases also PS isomers with the same DS, but the exact position of the substituents in such isomers remains elusive [71, 72]. A recent NMR study employed various ^1^H‐ and ^13^C‐NMR spectroscopic techniques, including correlated spectroscopy (COSY) and total correlation spectroscopy (TOCSY) measurements as well as ^1^H–^13^C heteronuclear single quantum coherence (HSQC) and heteronuclear multiple bond correlation (HMBC) experiments to characterize the distribution of the substituents in randomly substituted 2‐hydroxypropyl‐β‐CD (HP‐β‐CD) [73]. After the assignment of the signals of all protons and carbons in the spectra, it was possible to characterize the distribution of the 2‐hydroxypropyl substituents in positions 2, 3, or 6 of the glucopyranose moieties based on the signals of the C‐2, C‐3, and C‐6 atoms as well as the α‐carbon atoms of the hydroxypropyl substituents in the respective positions. Although this approach provided a valuable addition to the characterization of the PS in HP‐β‐CD batches, the composition with regard to the PS in individual CD isomers as well as the mixture of substitution isomers still remains unknown.

**FIGURE 2:**
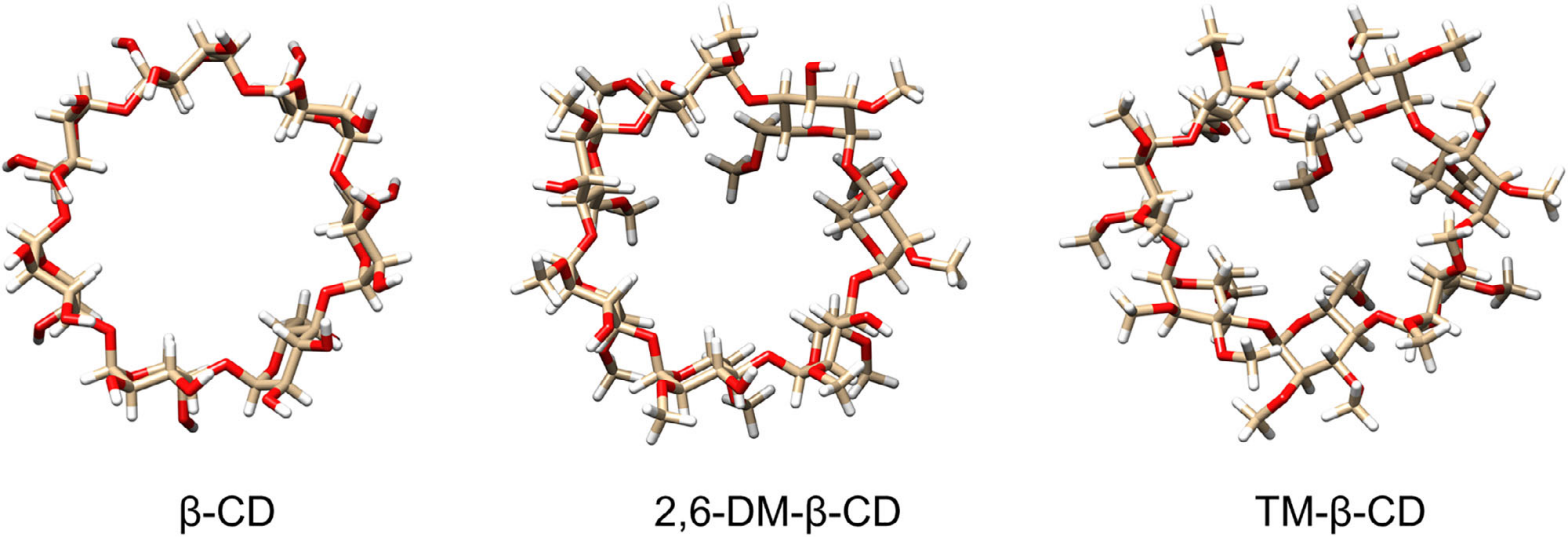
X‐ray structures of β‐CD, 2,6‐DM‐β‐CD, and TM‐β‐CD (extracted from the Cambridge Structural Database entries AGAZOX [75] for β‐CD, DEZMIE10 [76] for 2,6‐DM‐β‐CD and GELKEN10 [77] for TM‐β‐CD; see also Ref. [74]). 2,6‐DM‐β‐CD, *heptakis*(2,6‐di‐*O*‐methyl)‐β‐CD; TM‐β‐CD, *heptakis*(2,3,6‐tri‐*O*‐methyl)‐β‐CD.

Derivatization of the hydroxyl groups can also change the shape of the CD molecule and, consequently, the shape of the cavity as illustrated in Figure 2 for 2,6‐DM‐β‐CD and *heptakis*(2,3,6‐tri‐*O*‐methyl)‐β‐CD (TM‐β‐CD) in comparison to β‐CD. In the case of β‐CD, the round shape is due to the stabilizing effect of intramolecular O(3)H…O(2)H hydrogen bonds, and the round structure is retained in 2,6‐DM‐β‐CD due to the presence of intramolecular O(3)H…O(2)Me hydrogen bonds. In contrast, TM‐β‐CD adopts an elliptic structure. A recent molecular modeling study on methylated single isomer β‐CDs concluded that derivatization of the 3‐hydroxyl groups increases the distance between the O(2) and O(3) atoms of the neighboring d‐glucopyranose and, thus, weakens or abolishes hydrogen bonds [74]. Thus, *heptakis*(3‐*O*‐methyl)‐β‐CD (3‐M‐β‐CD), *heptakis*(2,3‐di‐*O*‐methyl)‐β‐CD (2,3‐DM‐β‐CD), *heptakis*(3,6‐di‐*O*‐methyl)‐β‐CD (3,6‐DM‐β‐CD), and TM‐β‐CD featured an elliptic shape, whereas hydrogen bonds are present in the 2‐ and/or 6‐methylated derivatives *heptakis*(2‐*O*‐methyl)‐β‐CD (2‐M‐β‐CD), *heptakis*(6‐*O*‐methyl)‐β‐CD (6‐M‐β‐CD), and 2,6‐DM‐β‐CD, stabilizing the round structure of these CDs.

An earlier study applied molecular dynamics simulations to study the effect of methylation on the structural features of β‐CD [78]. It was shown that a change of the conformation of one glucopyranose molecule within the CD can drastically modify the overall structure. Furthermore, upon crystallization of TM‐β‐CD from hot water, one of the glucopyranosyl units was found to be in the fully inverted ^1^C_4_ chair conformation [79], and a guest‐induced conformational change of one glucopyranosyl moiety to the ^0^S_2_ twist boat conformation was detected upon complexation of *m*‐iodophenol by TM‐β‐CD [77]. Distorted glucopyranose units were also found in the crystal structures of peracylated CDs [80]. Such data indicated that the derivatization of the hydroxyl groups increases the flexibility of the CD macrocycle. Overall, a CD may change the structure upon solute complexation by small changes of the torsion angles of the pyranose rings to accommodate the guest molecule [20].

In light of the presence of at least several tens of thousands of unique structures in a randomly substituted CD, Anderson et al. have suggested a universal nomenclature for individual structures, which has been derived in analogy to protein nomenclature [66]. The suggested nomenclature considers the substitution type and pattern, including cyclic permutations and covalently linked CD oligomers. In addition, a web‐based tool has been developed for automatic assignment of names to CD monomers [66].

CDs and derivatives derived from starch are composed of d‐glucopyranose units. The respective enantiomeric CDs composed of α‐1,4 linked l‐glucopyranose moieties have not been available until recently when the groups of Stoddart and Armstrong synthesized α‐l‐CD, β‐l‐CD, and γ‐l‐CD from l‐glucose‐derived building blocks [81]. The l‐CDs were subsequently evaluated for their applicability as chiral selectors in CE, and as expected, reversed EMO was observed as compared to the presence of the respective CDs composed of d‐glucopyranose units as chiral selectors in the BGE [82, 83].

### 2.2 Complex Formation and Complex Structure

Complexation of solutes by CDs occurs via formation of non‐covalent intermolecular interactions as summarized, for example, in Refs. [22, 27, 42, 43, 44, 45]. Because of the hydrophobicity of the CD cavity, inclusion of a solute is mediated via hydrophobic interactions within the cavity as well as hydrogen bonds with polar groups on the CD rims. Van der Waals forces can also contribute to formation of the complexes. Furthermore, due to the high electron density and Lewis base character originating from the direction of the nonbinding electron pairs of the glycosidic oxygens toward the interior of the cavity, dipole–dipole interactions and, in the case of aromatic solutes, π–π interactions can be established between CDs and guest molecules. Charged CDs can establish ionic interactions. Furthermore, conformational changes within the CD (induced fit) upon solute complexation can contribute to the complexation process.

In addition, complex formation is driven by the displacement of water molecules from the cavity [58, 84, 85]. Various amounts of water have been reported for CDs. For example, an average of 2.6 water molecules in the cavity of α‐CD, 6.5 water molecules in the case of β‐CD and 8.8 molecules for γ‐CD have been found on the basis of x‐ray diffraction data [58]. Dudev et al. have concluded from theoretical considerations and experimental data that α‐CD can accommodate up to 6 water molecules in the cavity [86], β‐CD up to 10 water molecules [87], and γ‐CD up to 7 water molecules [88]. The higher number of water molecules in the cavity of β‐CD as compared to the other CDs was attributed to the fact that the water molecules form a cluster between themselves with only negligible interactions with β‐CD in contrast to the other CDs [87]. Although there seems to be some controversy about the actual amount of water inside the cavity, these water molecules are considered “unorganized” (energetically frustrated) because only a lower number of hydrogen bonds can be established, in contrast to the average four interactions in bulk water. Thus, the expulsion of the water molecules into the bulk phase results in the fact that CD‐analyte complexation is an enthalpically driven process [58].

**FIGURE 3:**
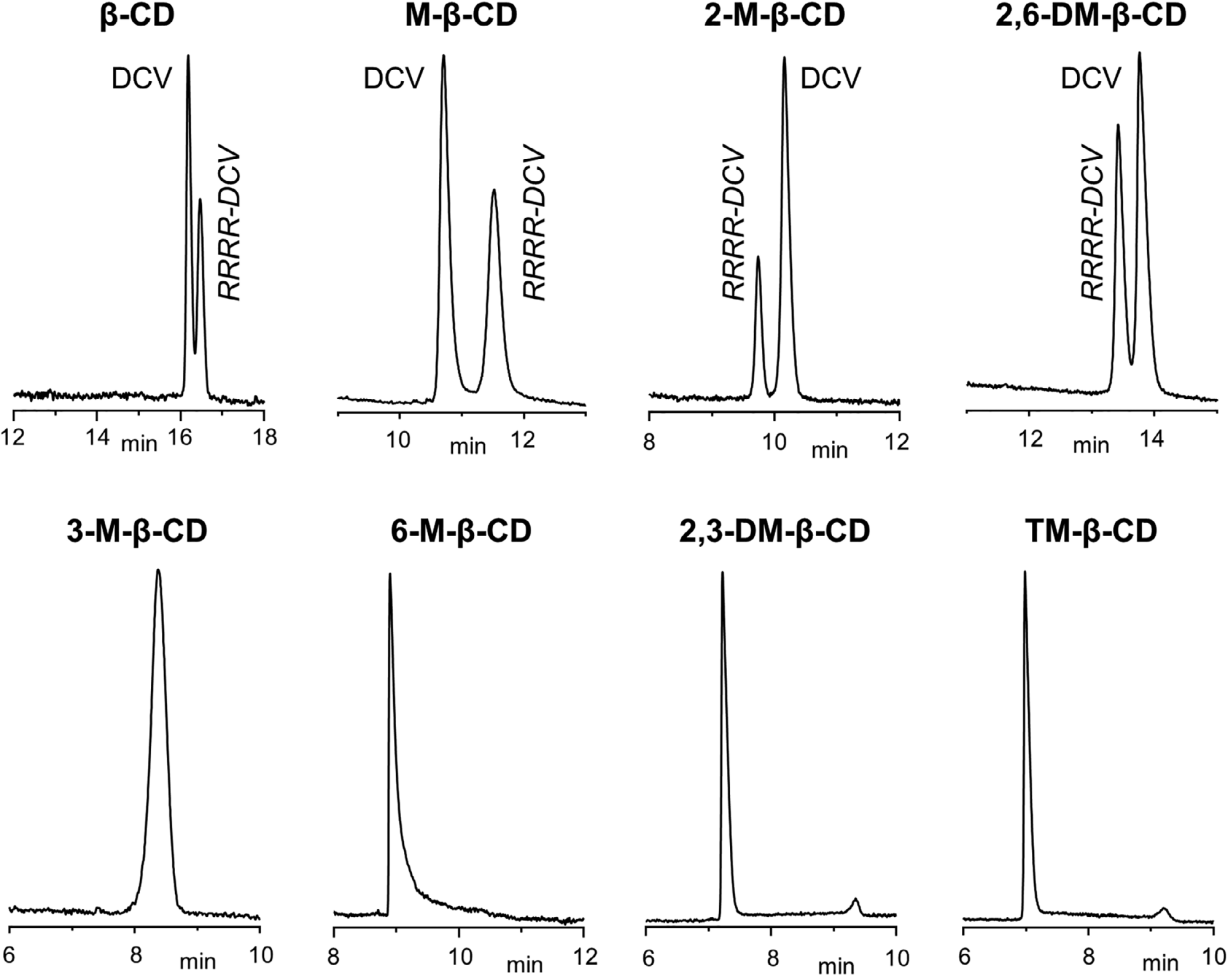
Electropherograms of the enantioseparation of a 2:1 mixture of DCV and *RRRR‐*DCV in the presence of β‐CD, M‐β‐CD (DS 7–14), and single‐isomer methylated β‐CDs. Experimental conditions: 40/50.2 cm, 50 µm i.d. fused‐silica capillary; 50 mM sodium phosphate buffer, pH 2.5; 20°C; 25 kV; CD concentration 7 mM, except β‐CD 60 mM (in the presence of 2 M urea) (see Refs. [72, 96]). 2,3‐DM‐β‐CD, *heptakis*(2,3‐di‐*O*‐methyl)‐β‐CD; 2,6‐DM‐β‐CD, *heptakis*(2,6‐di‐*O*‐methyl)‐β‐CD; 2‐M‐β‐CD, *heptakis*(2‐*O*‐methyl)‐β‐CD; 3‐M‐β‐CD, *heptakis*(3‐*O*‐methyl)‐β‐CD; 6‐M‐β‐CD, *heptakis*(6‐*O*‐methyl)‐β‐CD; M‐β‐CD, methylated β‐CD; TM‐β‐CD, *heptakis*(2,3,6‐tri‐*O*‐methyl)‐β‐CD.

Many studies have been performed combining various experimental techniques in order to determine the structures of CD–solute complexes in an attempt to rationalize chromatographic or electrophoretic enantioseparations. These techniques comprised primarily spectroscopic techniques, specifically NMR techniques, including experiments allowing the determination of the proximity of atoms or functional groups of CDs and solutes in the complexes, such as nuclear Overhauser enhancement spectroscopy (NOESY) or rotating frame Overhauser enhancement spectroscopy (ROESY). Especially, the combination of CE and NMR spectroscopy has been illustrative, because CDs can be studied in solution under comparable experimental conditions by both techniques [40, 89, 90, 91]. This is not necessarily true in case of chromatography, where the CD is fixed to a solid support, which may impair the accessibility of the cavity via either the secondary or the primary side. In addition, molecular modeling approaches have been frequently applied for understanding the chiral recognition mechanism, visualization of the complex structures, and estimating the thermodynamics of the complexation process as summarized in Refs. [41, 92, 93, 94, 95]. The advancement in computational hardware and software has made it relatively feasible to determine such data. However, a frequent error made in this context is the relatively uncritical application of molecular modeling. As mentioned above, attachment of a CD to a solid support may hinder the accessibility of the cavity so that modeling data obtained with the “free” CD do not correctly reflect the situation in chromatography. Another example is the use of modeling in the case of randomly substituted CDs. As pointed out, these CDs are a mixture of a large number of substitution and positional isomers. However, molecular modeling is often performed utilizing a CD isomer with a defined structure, that is, substitution pattern, which does not reflect the real isomeric composition of the randomly substituted CD. Several studies have described different EMO depending on the DS or substitution pattern of CDs. A recent example is the application of single‐isomer methylated β‐CDs (M‐β‐CDs) and randomly M‐β‐CD to the enantioseparation of the *SSSS*‐configured daclatasvir (DCV) and its enantiomer *RRRR*‐DCV as shown in Figure 3 [72, 96]. Randomly substituted M‐β‐CD with a DS 7–14 yielded the EMO DCV > *RRRR*‐DCV. The same EMO was observed for native β‐CD, whereas the application of 2‐M‐β‐CD and 2,6‐DM‐β‐CD resulted in the opposite EMO *RRRR*‐DCV > DCV. No enantioseparation was observed for 3‐M‐β‐CD and 6‐M‐β‐CD. In the presence of 2,3‐DM‐β‐CD and TM‐β‐CD, two peaks with a plateau in between were observed. This situation does not represent an enantioseparation [74, 96], but rather a slow complex dissociation phenomenon was also observed for DCV in the case of γ‐CD as chiral selector [97, 98]. Furthermore, the EMO of the DCV enantiomers depended on the DS of M‐β‐CD [72]. Derivatives with an isomeric purity with regard to 2,6‐DM‐β‐CD of at least 75% showed the same EMO as pure 2,6‐DM‐β‐CD, whereas M‐β‐CDs from different commercial sources with a lower content of 2,6‐DM‐β‐CD and with a DS varying between 7–14 and 12–16 resulted in the opposite EMO (DCV > *RRRR*‐DCV). This example clearly illustrates that a specific isomer of a CD does not adequately represent the nature of a randomly substituted CD containing numerous isomers, and conclusions drawn from one type of CD to another can be misleading. As mentioned above, a randomly substituted CD can be composed from several tens of thousands to about two millions of individual structures [66]. Despite these facts as well as other examples in the literature, a number of studies applied randomly substituted CDs in a CE enantioseparation and performed modeling with a structurally defined CD derivative. Even if using a single‐isomer CD for modeling resulted in the same EMO as observed in the CE experiments applying randomly substituted CDs, conclusions from such modeling data on the situation observed in CE experiments should be drawn with extreme care, as, in principle, such data should be considered conceptually erroneous.

Table 1 summarizes recent studies published between 2023 and early 2026 applying spectroscopic and/or molecular modeling techniques for the determination of the structures of CD–solute complexes in order to rationalize enantioseparations obtained by CE. For summaries of earlier studies, see, for example, [20, 22, 27, 41, 42, 43, 44, 45].

**TABLE 1:** Examples of studies combining CE enantioseparations with further techniques for the elucidation of the CD–solute complex structures.

Analyte	CD^a^	Spectroscopic techniques	Modeling techniques	Ref.
Acenocoumarin	γ‐CD	ROESY‐NMR	Molecular docking	[99]
Amlodipine	CM‐β‐CD	—	Molecular docking, DFT calculations	[100]
Benzyltetrahydroisoquinoline alkaloids	SBX, SGX	ROESY‐NMR	—	[101]
Cathinones	6‐M‐β‐CD, Succ‐β‐CD, SBX, 6‐SBE‐β‐CD	ROESY‐NMR	—	[102]
Chlorpheniramine, brompheniramine, and dimetindene	TM‐β‐CD	—	Molecular docking	[103]
Coumatetralyl	β‐CD	—	Molecular docking	[104]
Daclatasvir	TM‐β‐CD, γ‐CD	—	Molecular docking, molecular dynamics simulations	[98]
Daclatasvir	β‐CD, 2‐M‐β‐CD, 3‐M‐β‐CD, 6‐M‐β‐CD, 2,3‐DM‐β‐CD, 2,6‐DM‐β‐CD, 3,6‐DM‐β‐CD, TM‐β‐CD	—	Quantum mechanics, molecular dynamics simulations	[74]
Phe, Tyr, Trp	S‐β‐CD	—	Molecular docking	[105]
Tetramisole	β‐CD, 2,3‐DM‐β‐CD, HDA‐β‐CD, HMA‐β‐CD	ROESY‐NMR	Quantum mechanics	[106]
Vildagliptin	α‐CD	ROESY‐NMR	Molecular docking, DFT calculations	[107]
Xylopinine	β‐CD, SBX	ROESY‐NMR	—	[108]

Abbreviations: 2,3‐DM‐β‐CD, *heptakis*(2,3‐di‐*O*‐methyl)‐β‐CD; 2,6‐DM‐β‐CD, *heptakis*(2,6‐di‐*O*‐methyl)‐β‐CD; 2‐M‐β‐CD, *heptakis*(2‐*O*‐methyl)‐β‐CD; 3,6‐DM‐β‐CD, *heptakis*(3,6‐di‐*O*‐methyl)‐β‐CD; 3‐M‐β‐CD, *heptakis*(3‐*O*‐methyl)‐β‐CD; 6‐M‐β‐CD, *heptakis*(6‐*O*‐methyl)‐β‐CD; 6‐SBE‐β‐CD, *heptakis*(6‐*O*‐sulfobutylether)‐β‐CD; CM‐β‐CD, carboxymethyl‐β‐CD; DFT, density functional theory; HDA‐β‐CD, *heptakis*(2,3‐di‐*O*‐acetyl)‐β‐CD; HMA‐β‐CD, *heptakis*(2‐*O*‐methyl‐3‐*O*‐acetyl)‐β‐CD; ROESY, rotating frame Overhauser enhancement spectroscopy; SBE‐β‐CD, sulfobutylether‐β‐CD; SBX, *heptakis*(6‐deoxy‐6‐(2‐carboxyethyl)thio)‐β‐CD (subetadex); SGX, *heptakis*(6‐deoxy‐6‐(2‐carboxyethyl)thio)‐γ‐CD (sugammadex); S‐β‐CD, sulfated β‐CD; TM‐β‐CD, *heptakis*(2,3,6‐tri‐*O*‐methyl)‐β‐CD.

^a^ Only the CD is listed, which was used for modeling and/or NMR investigations of the solute–CD structure.

Primarily 1:1 (analyte:CD) complexes were observed in the cited studies except for the complexation of the antiviral drug daclatasvir (DCV) by γ‐CD, where a rare stoichiometric ratio of 2:1 was formed predominantly, whereas a 1:1 complex was present at much lower abundance [98]. Isothermal titration calorimetry measurements revealed the association of DCV in aqueous solutions, and subsequent molecular dynamics simulations indicated the formation of DCV dimers, which were subsequently complexed by γ‐CD with the biphenyl moieties of the DCV molecules inserted into the CD cavity. The terminal methyloxycarbonyl‐valine side chains protruded from the cavity “locking” the dimer inside. In the minor 1:1 complex, a DCV molecule was included into the cavity of γ‐CD in an analogous manner, but the single molecule was much more mobile. It was concluded that the complexation process of DCV and γ‐CD was dominated by a slow dissociation of the 2:1 complex, resulting in the observation of two peaks with a plateau between them in CE experiments. This was not related to an enantioseparation because identical observations were made when only DCV was analyzed in the presence of γ‐CD [97]. A slow dissociation equilibrium was also assumed as the reason for observing a plateau between peaks for DCV when TM‐β‐CD was present in the BGE [98], although in this case a 1:1 complex was derived for DCV and the CD [96].

**FIGURE 4:**
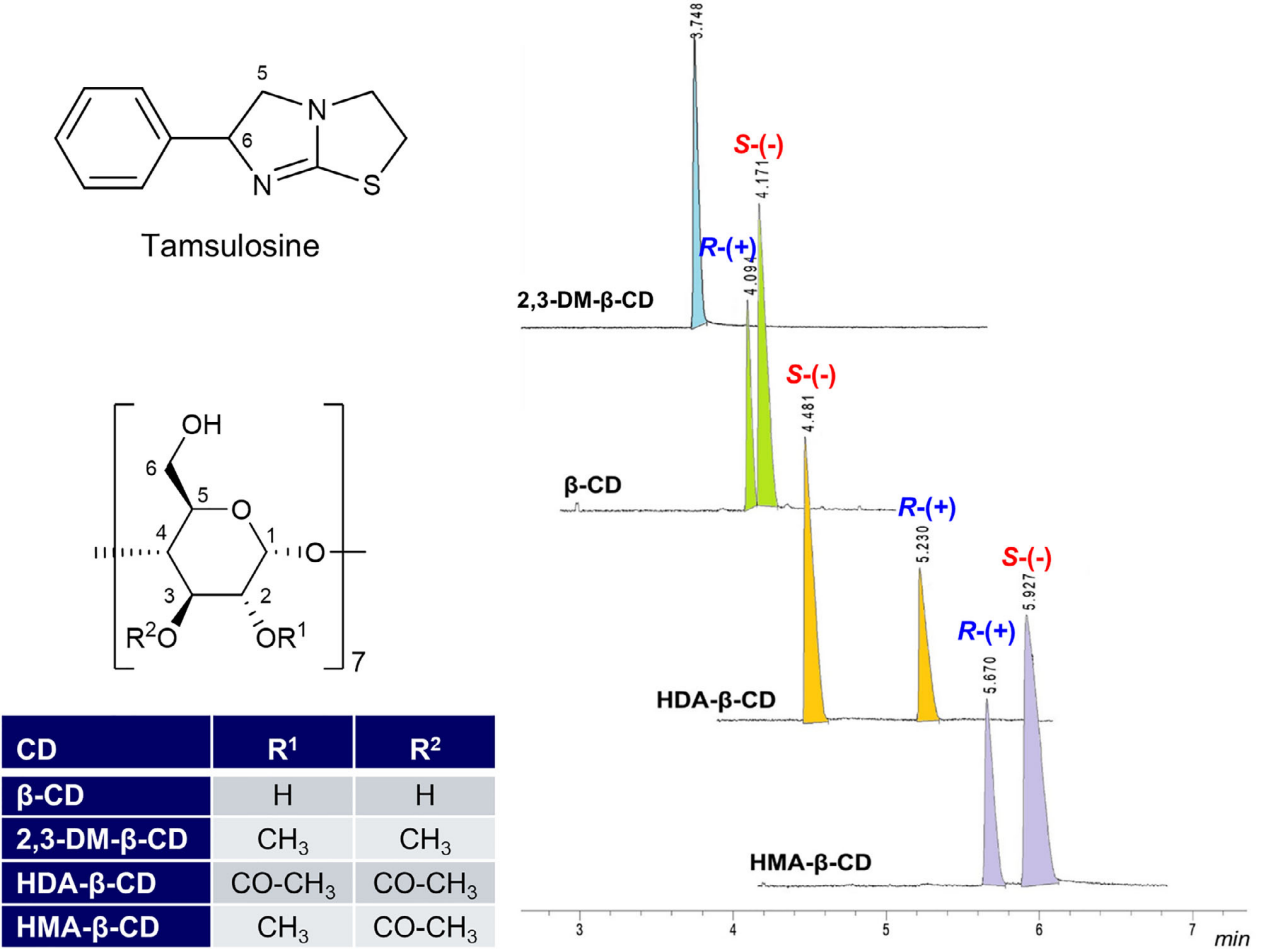
Structures of tetramisole and β‐CD derivatives and electropherograms of CE analyses in the presence of 2,3‐DM‐β‐CD, β‐CD, HDA‐β‐CD, and HMA‐β‐CD at a CD concentration of 17.6 mM. Experimental conditions: 24.5/32 cm, 50 µm i.d. fused‐silica capillary; 100 mM phosphoric acid adjusted to pH 3.0 with triethylamine; 20°C; 15 kV; 220 nm. 2,3‐DM‐β‐CD, *heptakis*(2,3‐di‐*O*‐methyl)‐β‐CD; HDA‐β‐CD, *heptakis*(2,3‐di‐*O*‐acetyl)‐β‐CD; HMA‐β‐CD, *heptakis*(2‐*O*‐methyl‐3‐*O*‐acetyl)‐β‐CD. Adapted from Ref. [106] with permission of Elsevier, 2025.

**FIGURE 5:**
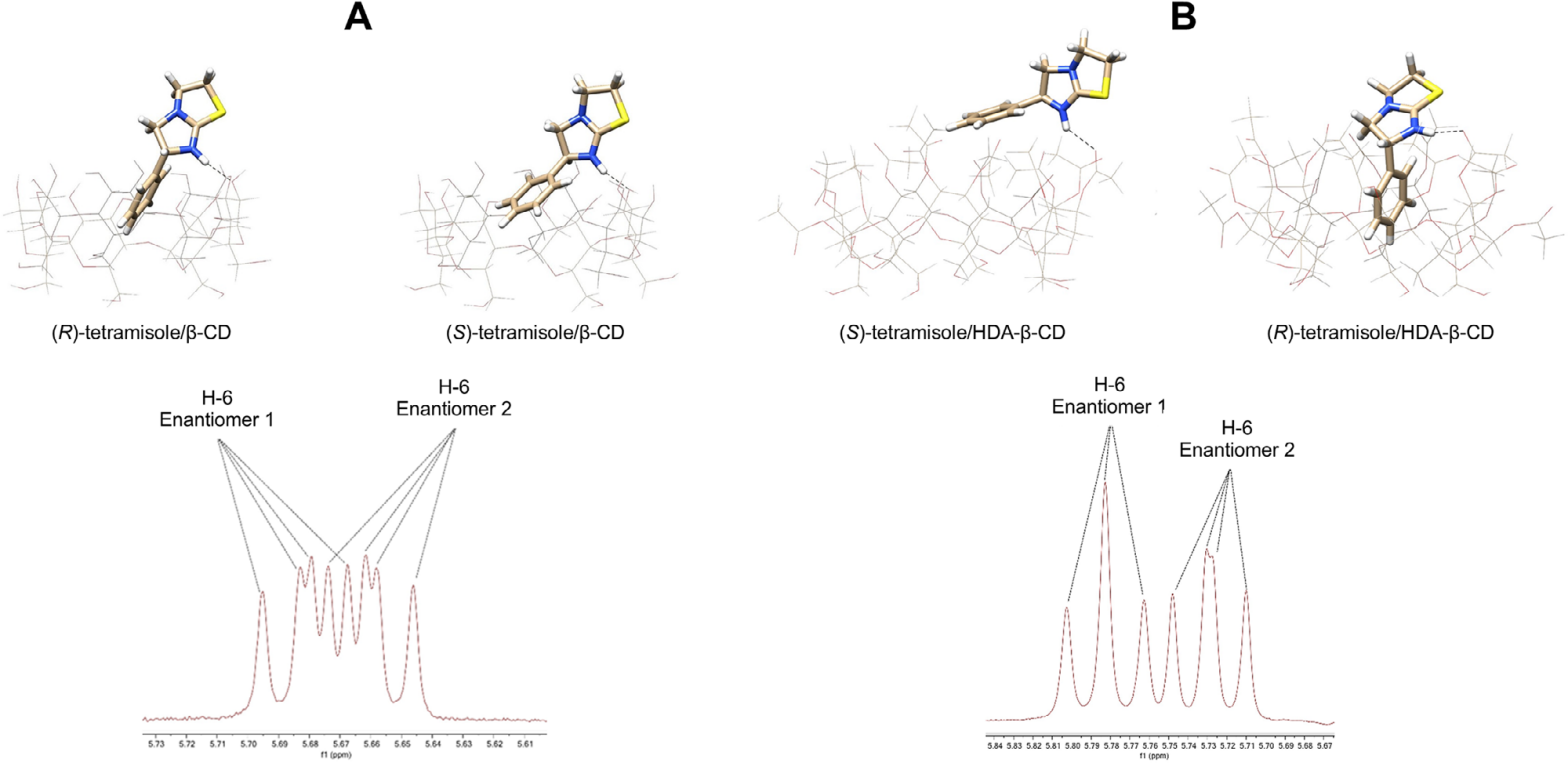
Expanded partial NMR spectra showing the signals of H‐6 of tetramisole in the complexes of racemic tetramisole with the CDs and optimized structures of the complexes of the CDs with the enantiomers of tetramisole: (A) β‐CD and (B) HDA‐β‐CD. The assignment of the structures of the HDA‐β‐CD complexes to the NMR signals were experimentally derived, the assignments in the case of the β‐CD complexes are tentative. HDA‐β‐CD, *heptakis*(2,3‐di‐*O*‐acetyl)‐β‐CD. Adapted from Ref. [106] with permission of Elsevier, 2025.

Although internal solute–CD complexes were observed in most cases, the formation of external complexes was also derived. An interesting example was observed when studying the separation of the enantiomers of tetramisole by β‐CD and the single‐isomer CDs 2,3‐DM‐β‐CD, *heptakis*(2,3‐di‐*O*‐acetyl)‐β‐CD (HDA‐β‐CD), and *heptakis*(2‐*O*‐methyl‐3‐*O*‐acetyl)‐β‐CD (HMA‐β‐CD) [106]. Enantioseparations were observed at pH 3.0 using a selector concentration of 17.6 mM for β‐CD, HDA‐β‐CD, and HMA‐β‐CD (Figure 4). In the case of 2,3‐DM‐β‐CD, a concentration of 112.7 mM was required to achieve an enantioseparation. The highest separation factor α was observed when using HDA‐β‐CD (*α* = 1.17). The EMO was (*S*) > (*R*) in the presence of HDA‐β‐CD and 2,3‐DM‐β‐CD, whereas it was (*R*) > (*S*) in the case of β‐CD and HMA‐β‐CD. Thus, either methylation or acetylation at both 2 and 3 positions apparently reversed the chiral recognition as compared to β‐CD or the “mixed” substation found in HMA‐β‐CD. NMR measurements performed on the tetramisole complexes with β‐CD and HDA‐β‐CD indicated splitting of the H‐6 proton at the stereogenic center of the analyte (Figure 5). However, although a doublet of doublets was observed for the H‐6 proton of both enantiomers in the presence of β‐CD (Figure 5A), a doublet of doublets was identified for H‐6 of one enantiomer in the presence of HDA‐β‐CD, whereas a triplet resulted for H‐6 of the other enantiomer (Figure 5B). A doublet of doublets indicated nonequivalence of the two H‐5 protons. This means that they are present in a chiral environment, that is, they interact with the CD. The triplet means that the respective protons are not in a chiral environment and, consequently, not inside the cavity. According to the NMR spectrum obtained with (*S*)‐tetramisole, the triplet refers to H‐6 of this stereoisomer. The results of the molecular modeling investigations (Figure 5) were in agreement with the NMR data. In the case of β‐CD, the optimized structures showed the insertion of the phenyl ring of both tetramisole enantiomers into the cavity via the wider secondary rim of the CD (Figure 5A). In contrast, only (*R*)‐tetramisole formed an inclusion complex with HDA‐β‐CD also entering the cavity from the secondary side, whereas (*S*)‐tetramisole formed only an external complex (Figure 5B), confirming the conclusions drawn from NMR data. Binding energies obtained by density functional theory (DFT) calculations were in agreement with the EMO seen in CE [106].

A similar observation of an inclusion complex for one enantiomer and external complexation of the other enantiomer was concluded for the coumarin‐derived anticoagulant acenocoumarin and γ‐CD [99]. 1D‐ROESY NMR data revealed intermolecular nuclear Overhauser effects (NOEs) between drug protons and H‐5 and H‐6 of the CD in the case of the (*S*)‐enantiomer, whereas no interactions were detected for the (*R*)‐enantiomer. Molecular docking located the (*S*)‐enantiomer inside the cavity with four hydrogen bonds and two hydrophobic interactions established between the solute and the selector. In contrast, (*R*)‐acenocoumarin interacted with the outside of the CD, establishing only three hydrogen bonds and one hydrophobic interaction. The stronger interaction of (*S*)‐acenocoumarin with γ‐CD compared to the (*R*)‐enantiomer was in agreement with the experimental EMO (*R*) > (*S*) [99].

Two studies applied randomly substituted CDs for enantioseparations in combination with molecular modeling. As discussed above, conclusions from such studies may be questionable because complexes obtained between the solutes and a defined CD isomer do not represent the multiple solute‐CD isomer complexes formed in the case of a randomly substituted CD. Thus, the separation of the enantiomers of amlodipine in the presence of carboxymethyl‐β‐CD (CM‐β‐CD) at pH 4.0 was modeled using the complexes with 6‐CM‐β‐CD [100]. Differences in binding energies and the type and number of established non‐covalent interactions were in agreement with the EMO, but the critical question of the single isomer CD being not representative for the randomly substituted CD was not addressed. In the second study, the enantioseparation of the aromatic amino acids Phe, Tyr, and Trp was achieved in the presence of S‐β‐CD in a phosphate buffer, pH 2.5, as BGE [105]. Modeling was performed with CDs containing 7, 14, and 21 sulfate substituents, that is, exclusively mono‐substitution in position 6 (7S‐β‐CD), di‐substitution in positions 2 and 6 (14S‐β‐CD), and the tri‐substituted isomer containing sulfate groups in positions 2, 3, and 6 (21S‐β‐CD). Differences in binding energies of the respective complexes all favored the stronger binding of d‐enantiomers in agreement with the EMO observed in CE. However, although not specifically addressed in the publication, different structures of the complexes were found depending on the substitution pattern. For example, in the case of Phe, the side chain was included in the cavity of 7S‐β‐CD, whereas the phenyl moiety penetrated the cavity in the case of 21S‐β‐CD. No significant inclusion was derived for 14S‐β‐CD with the amino acid positioned on top of the narrower primary side of the CD. Again, this illustrates that different structures of solute–CD complexes result depending on the substitution pattern of the CD and that a single structure does not represent the complex mixture of CD isomers in randomly substituted CDs.

A trend in recent years has been the combination of CDs with (chiral) ionic liquids (ILs) or deep eutectic solvents (DES) for enantioseparations, typically resulting in increased resolution compared to the application of a CD alone [109, 110, 111, 112, 113, 114, 115]. From a principal standpoint, ILs and DES share similarities but also differences [116]. Both are composed of anions and cations and are liquid at or close to room temperature (up to about 100°C). However, they differ in their starting materials and their preparation. ILs are composed of organic cations and organic or inorganic anions, whereas DES are a combination of Lewis bases (hydrogen bond acceptors) and Lewis acids (hydrogen bond donors). In addition, the synthesis of ILs may involve several steps, reagents as well as organic solvents and requires long reaction times. DES are prepared by (short‐term) heating or mechanical grinding of the two components until a homogenous liquid is formed. For further details on ILs and DES, see [116]. In the case of the application to analytical enantioseparations often either a single component or both components were chiral molecules.

The separation mechanism in chiral CE in the presence of ILs or DES has not been completely established. Evidence accumulated so far indicate a modification of the electroosmotic flow as one of the reasons for resolution improvement. However, often the effect is more pronounced in the case of ILs or DES containing chiral components as compared to the non‐chiral analogs, so that participation in the formation of a ternary complex or at least interaction between the selector–selectand complexes and components of the ILs or DES appear likely.

**FIGURE 6:**
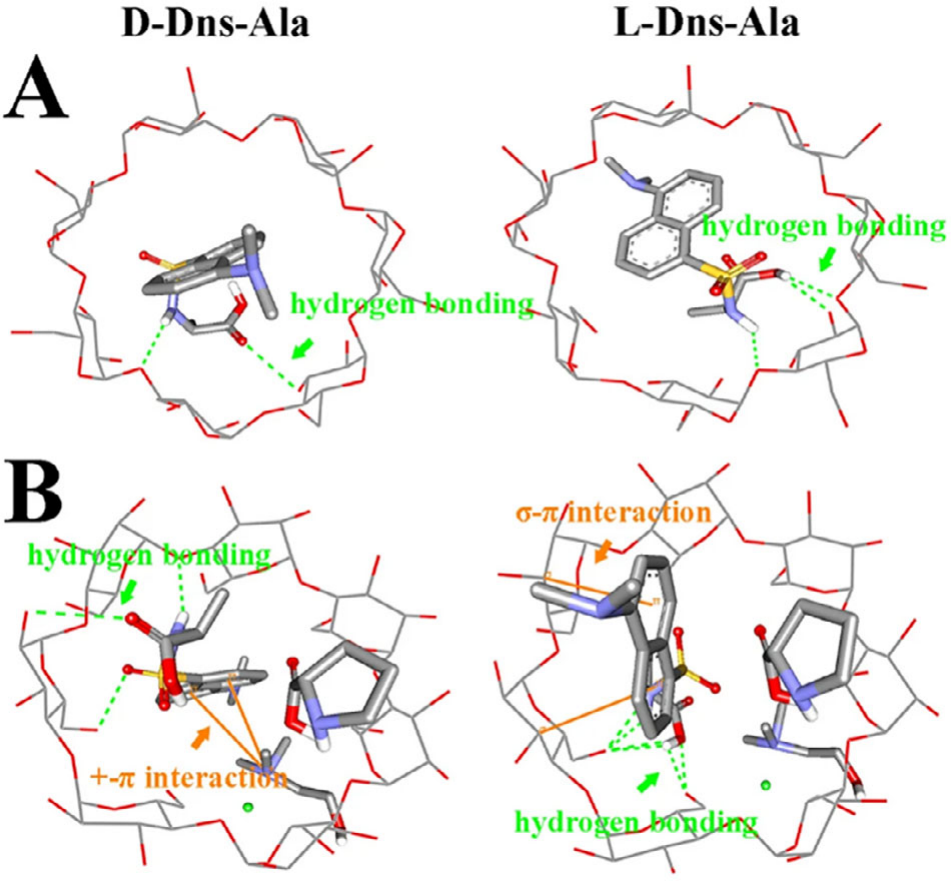
Molecular modeling configurations of Dns‐Ala enantiomers (A) in the presence of β‐CD and (B) in the presence of β‐CD and the choline/l‐Pro DES. Dashed green line, hydrogen bond; solid orange line, π‐interaction. C gray, H white, O red, N dark blue, S yellow, Cl, green. Dns, dansyl. Reproduced with permission from Ref. [117] Springer‐Nature 2026.

The enantioseparation of Dns‐amino acids in nonaqueous CE using β‐CD and amino acid‐derived DES as chiral selectors has been studied yielding increased migration times and higher resolution in the presence of a DES [117]. Δ*G* values obtained from molecular docking experiments showed higher values for the ternary system indicating stronger complexation in the presence of the DES. The molecular modeling structure of the complexes between β‐CD and the enantiomers of Dns‐Ala in the presence of a choline/l‐Pro DES indicated the interaction of the DES with the complex as shown in Figure 6. In the dual system (Figure 6B), additional hydrogen bonds as well as π‐interactions were established as compared to β‐CD alone (Figure 6A). In the case of d‐Dns‐Ala, direct interactions between the amino acid‐derived DES and the analyte were noted.

Studying the chiral CE separation of basic drugs by the dual system of SBE‐β‐CD and a betaine/urea DES, it was noted that superior resolutions were obtained for the enantiomers of primaquine by the dual system, whereas better enantioresolution was observed for terbutaline when only SBE‐β‐CD was present [118]. Molecular docking experiments were performed using the single isomer 6‐SBE‐β‐CD. In the case of primaquine, differences in the free binding energies (ΔΔ*G*) were larger with the dual system as compared to the absence of the DES, whereas the situation was opposite in the case of terbutaline in agreement with the CE results. In addition, in the absence of the DES, the enantiomers of prilocaine exhibited similar binding position, and interaction patterns with 6‐SBE‐β‐CD, but distinctly different inclusion geometries within the CD cavity in the presence of the DES consistent with the differences in ΔΔ*G* values observed for both systems. Although not discussed in the publication, significant differences in complex structures were also observed for terbutaline and the dual system, which is not necessarily in agreement with the ΔΔ*G* values. The authors correctly noted that commercially available SBE‐β‐CD consists of a distribution of substitution degrees rather than a single well‐defined molecular structure so that their docking analysis using 6‐SBE‐β‐CD did not reproduce the real separation environment quantitatively but rather provided a comparative and visual evaluation of enantioselective interaction trends [118].

A further novel approach for CD‐derived chiral selectors is the so‐called supramolecular DES (SUPRADES), which are considered a subclass of DES combining properties of DES and CDs [119]. In a SUPRADES, the CD acts as hydrogen bond acceptor that is combined with an organic acid, such as citric acid, lactic acid, or levulinic acid, as hydrogen bond donor. The enantioseparation of basic pharmaceutical drugs was significantly increased when applying a SUPRADES composed of β‐CD, M‐β‐CD or HP‐β‐CD, and l‐lactic acid compared to the individual components [120]. Docking studies using terbutaline as model compound revealed ternary inclusion complexes with the terbutaline enantiomers typically also interacting with l‐lactic acid. Additional interactions in the case of the SUPRADES were noted compared to the respective CD alone. Thus, the authors concluded that a SUPRADES may enhance enantioselectivity by amplifying the interactions between the drug and the CDs [118].

NMR experiments have also been employed for studying the interaction mechanisms between solutes, CDs, and ILs or DES. For example, the combination of CM‐β‐CD with a chiral IL composed of tetramethylammonium and d‐10‐camphorsulfonic acid resulted in superior resolution of the enantiomers of basic drugs with a large structural variety as compared to the use of CM‐β‐CD alone [121]. NMR experiments, including ROESY measurements, were performed using mirtazapine as model analyte. Shifts of the signals of aromatic and aliphatic protons close to the stereogenic center of mirtazapine as well as shifts of signals of d‐10‐camphorsulfonic acid indicated that both interacted with CM‐β‐CD so that formation of a ternary complex between the components was concluded. ROESY data indicated inclusion of an aromatic moiety of mirtazapine into the CD cavity. Furthermore, spatial proximity was noted between mirtazapine and d‐10‐camphorsulfonic acid. Analogous observations were obtained for the interaction between homatropine and the SUPRADES HP‐β‐CD/l‐lactic acid [122]. 2D NOESY measurements indicated the formation of a ternary complex with the heptatomic ring of the drug embedded in the cavity of HP‐β‐CD and spatial proximity between l‐lactic acid and the CD.

Furthermore, polymeric cations were used to form polymeric DES (PDES) with CM‐β‐CD [123]. These PDES self‐assembled in buffer solutions to form composite nanoparticles. The interactions between Phe as solute and the system composed of poly(diallyldimethylammonium), glycerol, and CM‐β‐CD were subsequently studied by NMR, including 2D NOESY NMR. Cross peaks revealed strong interactions between the PDES and CM‐β‐CD as well as inclusion of the aromatic ring of Phe into the CD cavity.

## 3 Enantioseparations of Pharmaceutical Drugs

Because stereoisomers of pharmaceutical drugs often differ in their pharmacological activity, toxicology, or pharmacodynamics, regulatory agencies, such as the Food and Drug Administration (FDA) or the European Medicines Agency (EMA), require the development of the active enantiomer of a drug candidate if the enantiomers differ qualitatively or quantitatively in their pharmacological or toxicological profiles. This has eventually led to the fact that in recent years only very few racemic drugs were approved, the vast majority being either single stereoisomer drugs or achiral substances [124, 125, 126]. Consequently, for quality control as well as drug development, effective and sensitive methods are required for the analysis of the stereochemical composition and purity of the drugs in synthetic batches, pharmaceutical formulations, and biological media. Although HPLC still remains the most frequently applied “workhorse” for this purpose [127], CE has proved to be an efficient technique, especially for hydrophilic and ionic compounds [22, 49, 51, 52, 54, 128, 129, 130, 131, 132]. Because of their availability and structural variability, pharmaceutical drugs are often used as model compounds for the evaluation of novel chiral selectors, including CDs, but such studies were not included in this overview. Instead, the following part will focus on the determination of the stereoisomeric purity of drugs in bulk substances or pharmaceutical preparations as well as enantioseparations of drugs and metabolites in biological matrices.

In the past decade, quality‐by‐design (QbD) principles have been increasingly applied to the development, optimization, and validation of analytical methods, including chiral separations by CE. QbD results in a rational approach as well as a well‐understood process regarding the effect of the method parameters on the analytical outcome based on science principles and risk management. Design‐of‐experiment (DoE) approaches using chemometric tools are an integral part of a QbD‐based method development, allowing the simultaneous investigation of experimental parameters as well as their interdependence. The previously applied one‐factor‐at‐a‐time (OFAT) approach does not yield conclusions on interactions of experimental parameters. The first step in analytical QbD is the definition of the analytical target profile (ATP) describing the method performance characteristics and associated criteria to ensure that the resulting data are fit for the intended purpose. A typical approach in QbD begins with an explanatory phase of selection of a chiral selector and experimental parameters, which are studied in their respective ranges. This initial phase is based on the experience of the analyst as well as properties of the analyte. Subsequent development includes the identification of the analytical procedure variables (the critical process parameters, CPPs), which affect the critical quality attributes (CQAs) of the performance of the method. Upon identification of the CQAs, DoE is performed to find the method operable design region (MODR), formerly termed design space, which characterizes the range of parameter settings where the ATP is met. As long as the parameters are selected within this region, robustness testing is not necessarily part of further method evaluation because within the MODR the ATP is met so that small variations of the experimental parameters will not significantly impact the outcome. After validation, the method is applied to sample analysis. The QbD approach for analytical methods also includes a method control strategy and life cycle management. QbD and DoE approaches in analytical method development, including stereoselective CE, have been reviewed, for example, in Refs. [133, 134, 135, 136, 137, 138, 139]. DoE approaches are also recommended by the guideline on analytical method development Q14 by the International Council for Harmonisation of Technical Requirements for Pharmaceuticals for Human Use (ICH) [140].

During the period of time summarized here, only a few studies were published on the analysis of the chiral purity of drug substances as listed in Table 2. Interestingly, in most cases randomly substituted CDs were employed [141, 142, 143, 144] and one study used a dual CD system [145]. The combination of selectors can be quite useful as demonstrated in many previous studies [47, 48]. Furthermore, QbD and DoE approaches were applied for method development in the majority of the investigations. Typically, the methods allowed the determination of 0.1% of the other enantiomer as a chiral impurity. In the case of tamsulosin, related substance impurities could be quantified simultaneously [145].

**TABLE 2:** Recent applications of enantioselective EKC in the analysis of the enantiomeric purity of drugs using CDs as chiral selectors.

Analyte	CD (concentration)	Background electrolyte	Comments	Ref.
Apremilast	Succ‐β‐CD (25 mM)	50 mM sodium phosphate, pH 7.0	Polybrene‐coated capillary, LOQ 0.10% (*R*)‐enantiomer	[141]
Duvesilib	SBE‐β‐CD (10 mM)	25 mM sodium formate, pH 3.0	LOQ 0.3% (*R*)‐enantiomer	[143]
Indacaterol	CM‐α‐CD (0.7%, m/v)	50 mM sodium formate, pH 4.0	DoE in method development, short end injection, LOQ 0.2 mg mL^−1^ (*S*)‐enantiomer	[144]
Silodosin	CM‐β‐CD (40 mg mL^−1^)	100 mM sodium phosphate, pH 2.9	DoE in method development, LOQ 0.07% (*S*)‐enantiomer	[142]
Tamsulosin	S‐β‐CD (40 mg mL^−1^) and CM‐α‐CD (7 mg mL^−1^)	30 mM sodium phosphate, pH 3.0	DoE in method development, LOQ 0.10% (*S*)‐enantiomer, simultaneous analysis of related substances	[145]
Vildagliptin	α‐CD (50 mM)	75 mM sodium acetate, pH 4.5	DoE in method development, LOQ 0.08% (*R*)‐enantiomer	[107]

Abbreviations: CM‐β‐CD, carboxymethyl‐β‐CD; CM‐α‐CD, carboxymethyl‐α‐CD; DoE, design of experiments; SBE‐β‐CD, sulfobutylether‐β‐CD; Succ‐β‐CD, succinyl‐β‐CD; S‐β‐CD, sulfated β‐CD.

A chiral CD‐based CE method has been developed for the analysis of racemic ketorolac in pharmaceutical formulations [146]. Using a BGE consisting of a 50 mM sodium phosphate buffer, pH 7.0, and 7 mM TM‐β‐CD, the enantioseparation was achieved with an applied voltage of 30 kV at a temperature of 15.7°C in less than 11 min. Analysis of an injection solution and eye drops confirmed the racemic nature of the drug in both preparations.

**FIGURE 7:**
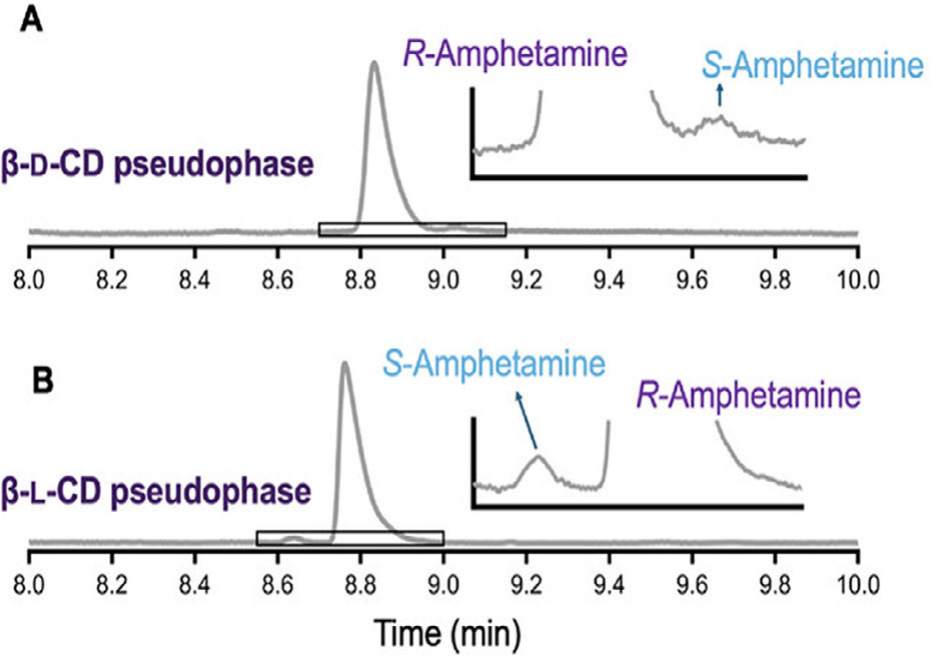
Electropherograms of enantioseparations of amphetamine using 15 mM (A) β‐d‐CD and (B) β‐l‐CD. The sample was prepared by the addition of 50 µL of (*R*)‐amphetamine solution (0.1 mg mL^−1^) to 1 µL of the (*S*)‐amphetamine solution (0.1 mg mL^−1^) resulting in ∼2% enantiomeric impurity. Experimental conditions: 42/52 cm, 50 µm i.d. fused‐silica capillary; 200 mM sodium phosphate buffer, pH 2.2; 25 kV; 25°C; 190 nm. Reproduced from Ref. [83] with permission from Wiley, 2026.

Considering the fact that impurities should be quantified at the 0.1% level, the EMO is an important issue in chiral analytical methods. It is always desirable to detect the minor impurity in front of the major peak of the parent compound, resulting in improved detection/quantitation limits and precision. Because CDs were available only with the d‐configuration of the glucopyranose moieties, this had been achieved by a variety of experimental conditions as summarized in Refs. [37, 147]. As mentioned above, β‐l‐CD has been synthesized recently as a mirror image of the natural β‐CD composed of d‐glucopyranose [81]. Systematic studies or studies employing validated methods for the determination of the chiral purity of drugs have not been conducted because of the limited quantities of β‐l‐CD currently available. Nonetheless, it has been demonstrated that application of β‐l‐CD results in reversal of the EMO with an improved detection of chiral impurities as illustrated for amphetamine in Figure 7 [83].

CD‐mediated enantioseparations have also been applied to the enantioselective analysis of drugs in biological fluids. The CE method developed for the analysis of the enantiomeric purity of duvesilib has also been used for the stereoselective analysis of the drug in serum and wastewater samples [143]. Preconcentration was carried out via the so‐called preconcentration micro solid‐phase extraction in a disposable needle technique. A sensitive CE method for the determination of the chiral barbiturates secobarbital and pentobarbital in body fluids was developed by integrating ultrasound‐assisted dispersive liquid–liquid microextraction with large‐volume sample stacking with polarity switching [148]. HP‐β‐CD and taurocholate were used as chiral selectors in a 600 mM Tris–boric acid buffer, pH 9.5. Under optimized conditions, a 6400‐fold increase in sensitivity was achieved with an LOD of 0.1 ng mL^−1^. The cationic CD 1‐butyl‐3‐β‐cyclodextrinimidazolium tosylate in a 25 mM sodium phosphate buffer, pH 6.4, has been employed for the enantioseparation of ketoprofen and ketorolac [149]. The method was validated for ketorolac and applied to the analysis in tablets and human plasma. Furthermore, the enantiomers of clenbuterol could be analyzed in dried urine spots by a CE–MS method using as BGE a 30 mM ammonium formate buffer, pH 3.4, containing 5.3 mM CM‐β‐CD in combination with sample stacking by electrokinetic injection [150]. The method was validated and applied to the analysis of a sample of a patient undergoing clenbuterol therapy. Although not applied to a practical study, a CE–MS method has been developed for the enantioselective analysis of tamsulosin [151]. Using a BGE composed of 200 mM ammonium acetate, pH 4.0, and 4.0 mg mL^−1^ S‐β‐CD in combination with online stacking preconcentration, an LOQ of 4.8 nmol L^−1^ and an LOD of 1.6 nmol L^−1^ could be achieved.

**FIGURE 8:**
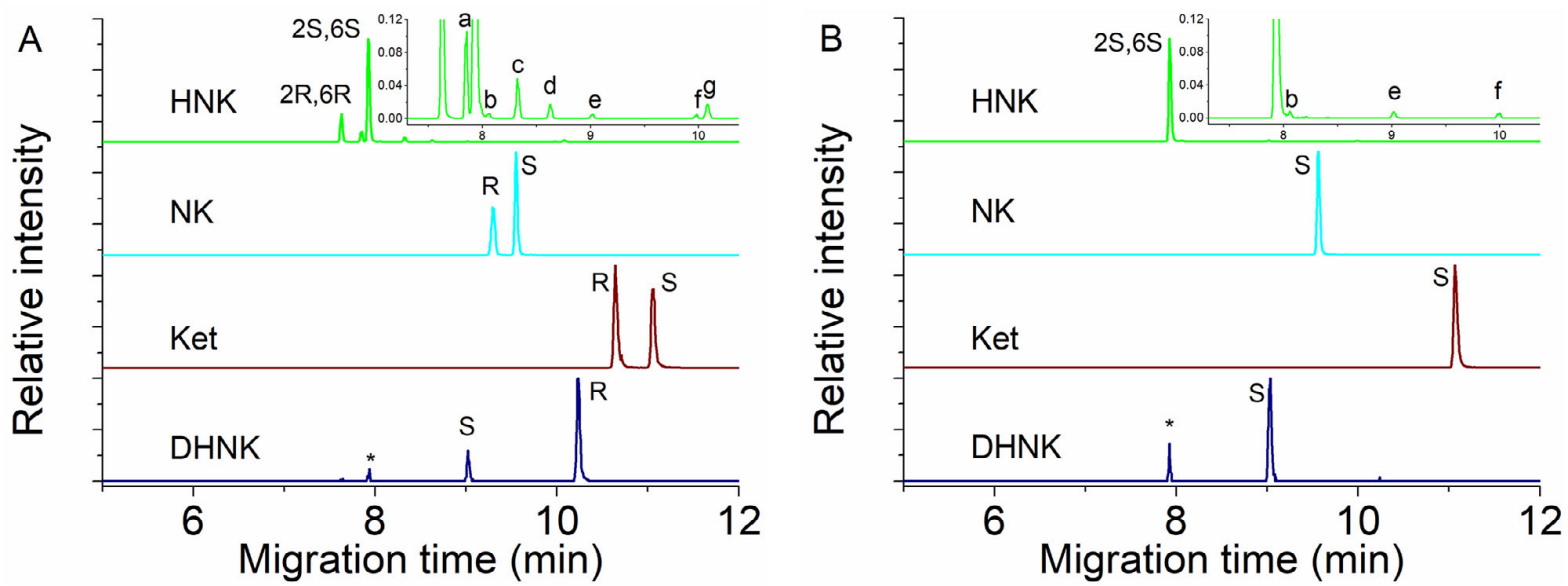
Extracted ion electropherograms of the CE–MS separation of the enantiomers of ketamine (Ket) as well as the metabolites norketamine (NK), dehydronorketamine (DHNK), and hydroxynorketamine (HNK) in rat plasma samples collected 50 min after administration of a dose of 10 mg kg^−1^ of racemic Ket (A) and (*S*)‐Ket (B). The separation was achieved in a 42.5 cm, 25 µm i.d. fused‐silica capillary with a covalent coating with poly(acrylamide‐*co*‐sodium‐2‐acrylamido‐2‐methylpropanesulfonate). BGE: 10 mM NH_4_OH, 104 mM AcOH, 10% (v/v) ethanol, apparent pH 3.75; 47 mm zone of S‐β‐CD (30 mg mL^−1^) and 55 mm zone of S‐γ‐CD (10 mg mL^−1^); 30 kV. The peaks (a–g) in the inserts correspond to unidentified HNK isomers. The peak marked with an asterisk in the extracted ion electropherogram of DHNK corresponds to HNK after water loss during the ESI process. Reproduced from Ref. [152] with permission Elsevier, 2025.

A CE–MS method combining a partial filling technique with a dual chiral selector system has been developed to study the metabolism of ketamine in rats [152]. The dual selector system consisted of two separate zones of 30 mg mL^−1^ S‐β‐CD and 10 mg mL^−1^ sulfated γ‐CD (S‐γ‐CD) dissolved in the BGE composed of 10 mM ammonium hydroxide, 104 mM acetic acid, and 10% (v/v) ethanol with an apparent pH of 3.75. Furthermore, the capillary was coated with a negatively charged polymer. Plasma extraction was performed by liquid–liquid extraction using dichloromethane. Under optimized conditions, the enantiomers of ketamine and its metabolites norketamine, dehydronorketamine, and 6‐hydroxynorketamine could be analyzed in a single run as shown in Figure 8 for rat plasma samples 50 min after administration of a dose of 10 mg kg^−1^ of racemic ketamine (Figure 8A) and (*S*)‐ketamine (Figure 8B). Upon administration of racemic ketamine, the (*S*)‐enantiomer was metabolized faster, with (2*S*,6*S*)‐norketamine as the major metabolite, whereas (2*R*,6*R*)‐norketamine as metabolite of (*R*)‐ketamine was detected at a significantly lower concentration. As expected, the concentration of the main metabolites with (*S*)‐configuration was approximately half the concentration when (*S*)‐ketamine was analyzed [152].

Another study used S‐γ‐CD as single chiral selector in CE–MS for the analysis of ketamine and metabolites in equine urine with a focus on the separation of the four stereoisomers of 6‐hydroxynorketamine [153]. Separations were performed in a fused‐silica capillary using a BGE composed of 5% (v/v) of a 20% (m/v) solution of S‐γ‐CD in 20 mM ammonium formate with 10% (v/v) formic acid. Partial filling of 75% of the capillary length provided the separation of the four stereoisomers of 6‐hydroxynorketamine, the stereoisomers of norketamine and dihydronorketamine as well as the detection of further low‐abundant metabolites. The method was subsequently applied to the analysis of equine urine. A sensitive assay for the enantioselective analysis of amlodipine in plasma, including acetonitrile‐driven field‐amplified sample stacking, was developed [154]. Separation was achieved in a fused‐silica capillary with a BGE containing 6.25 mM sodium borate and 25 mM sodium dihydrogen phosphate, adjusted to pH 2.5 by the addition of phosphoric acid, and 30 mg mL^−1^ HP‐β‐CD. Calibration was performed for the concentration range of 0.2475–19.80 ng mL^−1^ for each enantiomer in plasma, and the validated method was subsequently applied to the analysis of (*S*)‐amlodipine in plasma of volunteers upon oral administration of a dose of 5 mg (*S*)‐amlodipine besylate tablets in comparison to a dose of 10 mg amlodipine besylate tablets.

Despite extensive studies on the enantioseparation ability of combinations of CDs with (chiral) ILs or DES, including SUPRADES using pharmaceutical drugs as model compounds, only few systems have been applied to “real” pharmaceutical analysis in the period of time considered here. Thus, the enantiomeric purity of (*S*)‐chlorpheniramine was determined using 30 mM sodium hydrogen phosphate, pH 3.5, containing 20% (v/v) methanol and 8 mM CM‐β‐CD and 20 mM of a tetramethylammonium/d‐10‐camphorsulfonic acid DES in a fused‐silica capillary applying amperometric detection [121]. Under optimized conditions, an LOQ of 5 µg mL^−1^ was achieved, and a relative content of 1.59% (*R*)‐chlorpheniramine was detected in (*S*)‐chlorpheniramine samples.

The combination of SBE‐β‐CD and a DES obtained from choline chloride and citric acid was applied to the enantioseparation of mirabegron [155]. Using a 50 mM sodium phosphate buffer, pH 3.0, containing 1.25% (w/v) SBE‐β‐CD and 2.0% (w/v) of the DES, the mirabegron enantiomers were separated with a resolution value of 2.0 in less than 12 min. The method was validated and subsequently applied to the analysis of the enantiomeric purity of (*R*)‐mirabegron. The relative LOD of the (*S*)‐enantiomer was 0.1% in a sample containing 300 mg mL^−1^ (*R*)‐mirabegron. In commercial tablets, the content of (*S*)‐mirabegron was below the detection limit. The method was further applied to analyze the racemic drug in spiked urine samples. A choline chloride/urea DES in combination with CM‐β‐CD was applied to the analysis of adrenaline in an injection solution as well as for the determination of racemic salbutamol in an inhalation preparation [156].

## 4 Concluding Remarks

Although CDs continue to be the most frequently applied chiral selectors in analytical enantioseparations by CE, the general interest in the application of CD‐mediated CE enantioseparations of pharmaceutical drugs to “real” samples appears to be somewhat diminished on the basis of the number of publications in recent years. A strong topic remain studies involving spectroscopic and modeling techniques in an attempt to understand the mechanisms underlying an enantioseparation.

As in the past, the most frequent use of chiral CE in pharmaceutical analysis appears to be the determination of the chiral purity of drug substances in bulk ware and formulations. The combination of CDs with ILs, DES as well as SUPRADES has been a relatively new research field yielding increased enantioresolutions in most cases. Although the cause of the increased resolution as well as the separation mechanism are not fully understood at present, these systems appear promising and are starting to be applied to the analysis of pharmaceutical products.

## Conflicts of Interest

The author declares no conflicts of interest.

## Data Availability

The author has nothing to report.
